# Supplementary figures and images for: Cellular heterogeneity map of diverse immune and stromal phenotypes within breast tumor microenvironment
Source: PeerJ. 2020 Jul 10;8:e9478. doi: 10.7717/peerj.9478 (PMC7357563; doi:10.7717/peerj.9478)

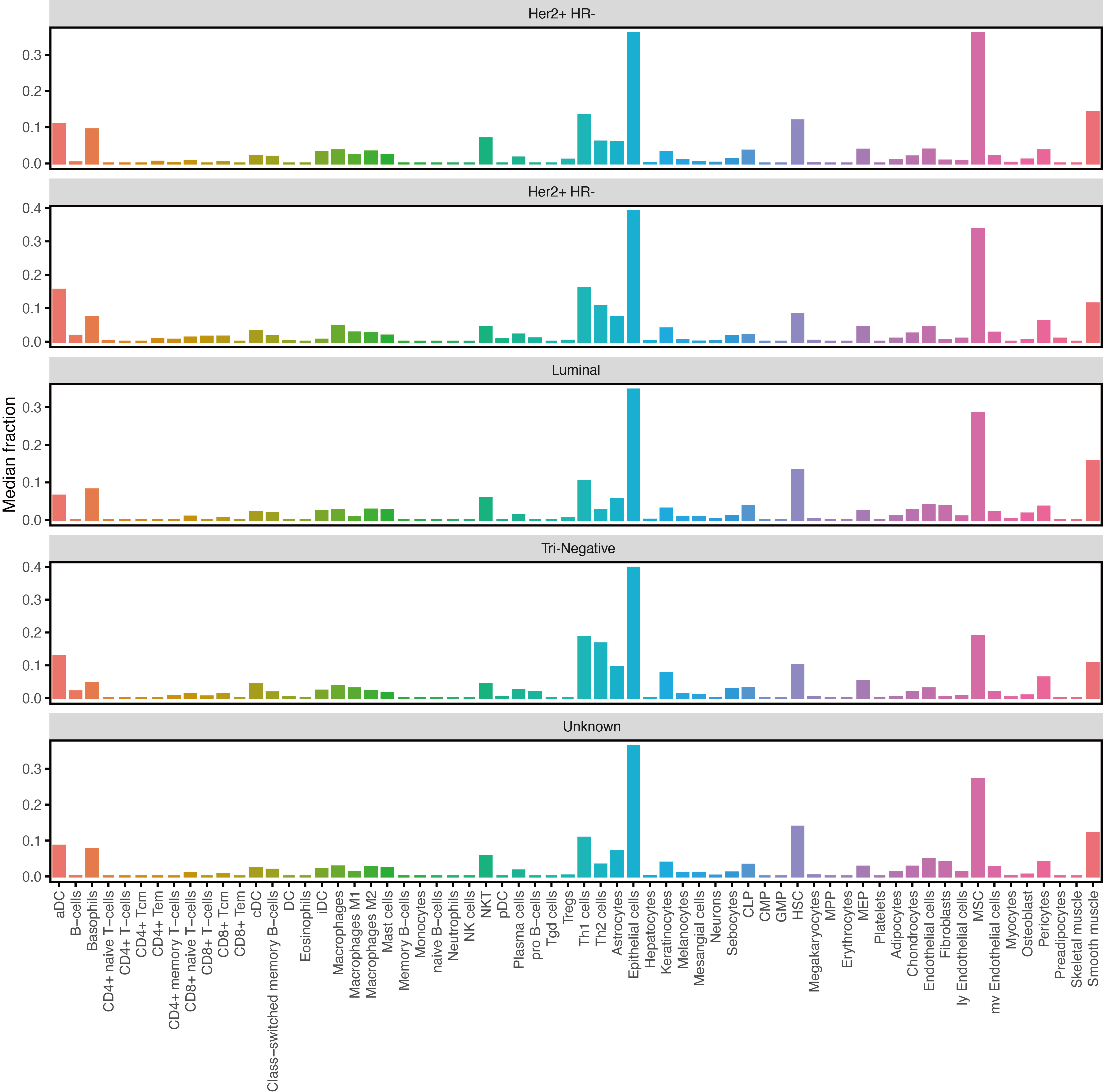

Supplement: Supplemental Information 1 [file peerj-08-9478-s001.jpg]

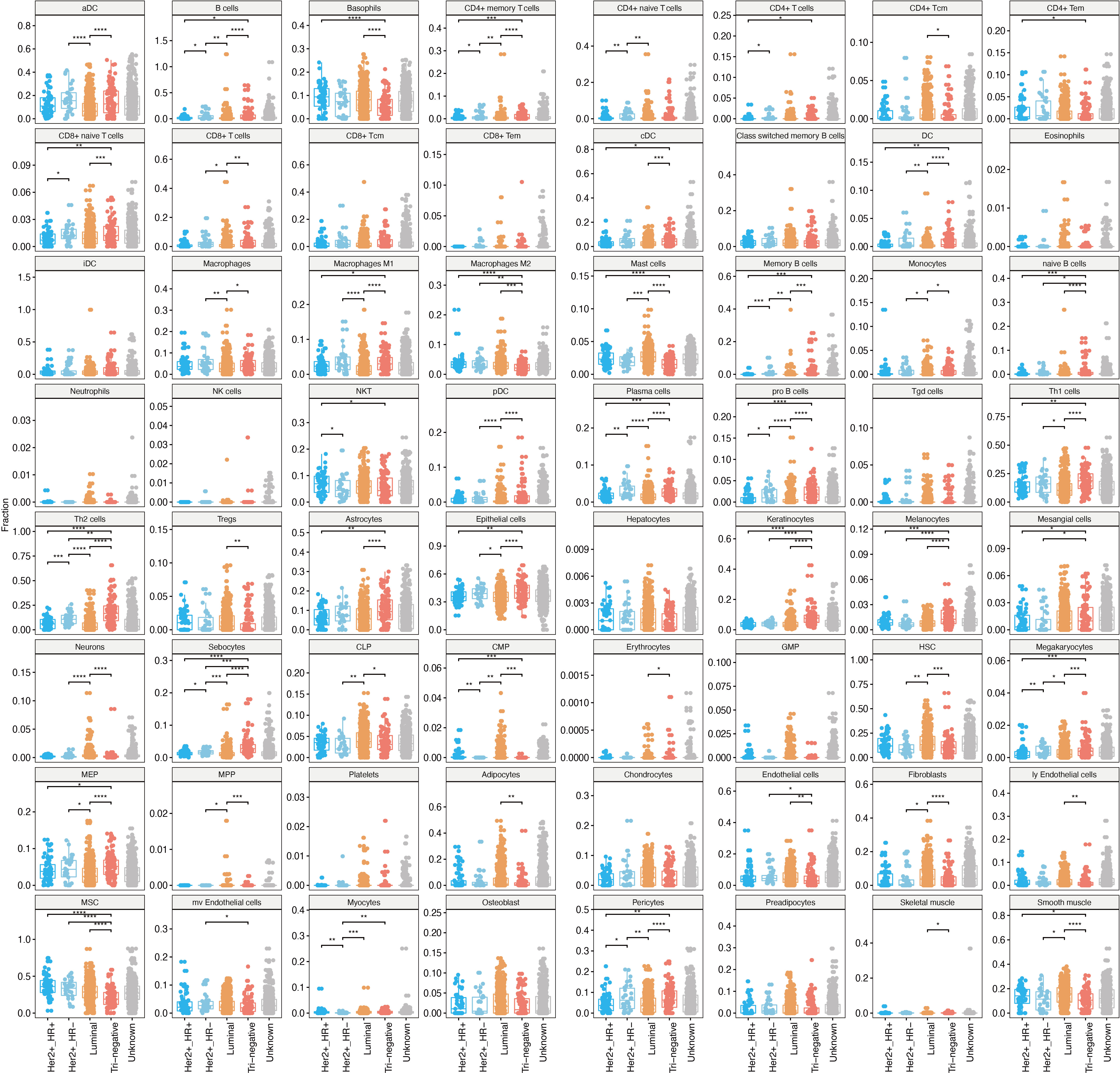

Supplement: Supplemental Information 2 [file peerj-08-9478-s002.jpg]
